# Formative Evaluation of Using Action Learning in a Master of Medical Education Assessment and Measurement Course

**DOI:** 10.7759/cureus.26523

**Published:** 2022-07-03

**Authors:** Arif M Rana, Harold Wiggin, Hilda DeGaetano, Jill Wallace-Ross, Robin J Jacobs

**Affiliations:** 1 Health Informatics and Health Information Management, State University of New York (SUNY) Polytechnic Institute, Albany, USA; 2 Health Informatics, Nova Southeastern University Dr. Kiran C. Patel College of Osteopathic Medicine, Fort Lauderdale, USA; 3 Medical Education and Simulation, Nova Southeastern University Dr. Kiran C. Patel College of Osteopathic Medicine, Fort Lauderdale, USA; 4 Family Medicine, Nova Southeastern University Dr. Kiran C. Patel College of Osteopathic Medicine, Fort Lauderdale, USA; 5 Research, Health Informatics, Medical Education, Nova Southeastern University, Fort Lauderdale, USA

**Keywords:** assessment, masters course, medical education, evaluation, curriculum, action learning

## Abstract

Background: The future success of any graduate or professional degree program is dependent upon continuous feedback provided by instructors and students. Various teaching models used by medical educators include didactics, problem/case-based learning, small/large group work, distance/online education, simulation, labs, and service/experiential learning. Action Learning is a process “that involves a small group working on real problems, taking action, and learning as individuals, as a team, and as an organization.” Medical school curricula usually begin with a mostly knowledge-based approach to learning the relevant science courses. While it may include some experiential learning, there is limited organized reflection. The idea inherent in Action Learning is “learn while doing” and “reflect on the experience.” This paper reports the process and outcomes of using the Action Learning Model (ALM) in teaching a master’s level assessment and measurement medical education class.

Objective: The objective of this quality improvement education study was to ascertain students’ knowledge, skills, and attitudes demonstrated in conducting substantive evaluations using the ALM in a graduate medical education assessment and measurement course.

Method: This study was a formative evaluation of a 16-week master’s level medical education assessment and measurement course. The curriculum included teaching the traditional knowledge, skills, and attitudes (KSAs) to conduct formative and summative evaluations in medical education. In addition, students learned applicable quality improvement skills. Specifically, they learned how to identify and work with valid customer (student) requirements, how to map and improve processes, and how to collect and analyze process data. Students were taught the KSAs while conducting a formative evaluation of the class as their major project. They evaluated the class they were taking while reflecting on the experience. In addition to the ALM, the course incorporated both the Bloom Taxonomy (a hierarchical framework for cognition and learning objectives) and the Kirkpatrick Model (a globally recognized method of evaluating the results of training and learning programs). The one-sample significance test was used to evaluate the median of the difference between the pre-and post-test groups. Descriptive statistics were also performed.

Results: Nine students who were medical students, dental students, physicians, and simulation lab technicians participated in the course. Students learned medical education assessment and measurement of knowledge, skills, and attitudes (KSAs) and experienced the process of performing a formative evaluation. The post-test results for all students combined revealed that 277 of the 450 (61.6%) data points were greater than zero. A total of 139 data points showed no improvement and 34 results were worse than the pretest.

Discussion: The ALM for teaching assessment and measurement in medical education can be challenging, but it may provide a more realistic and rewarding educational experience. The students gained a greater appreciation of the positive and negative aspects of using an experiential approach. Finally, the weekly formative surveys provided regular feedback that led to instructional improvements. With regards to medical education, action learning is best suited for students during the clinical phase of their education.

## Introduction

The future success of any graduate or professional degree program is dependent upon continuous feedback provided by course instructors and students. University and college administrators, faculty, and researchers strive to provide quality education for students and employ different techniques to do so. Various models of teaching are used in medical school curricula, with the predominant ones being didactics, problem/case-based learning, small/large group work, distance/online education, simulation, labs, and service/experiential learning [1].

Action Learning is a process “that involves a small group working on real problems, taking action, and learning as individuals, as a team, and as an organization” [2]. It helps organizations develop creative, flexible, and successful strategies for pressing problems. Developed in the early 1980s by Reg Revans, action learning has been well received by the business/corporate world [3]. However, it has yet to be adopted by the health professions academic society. Action learning may be, in fact, in explicit opposition to the didactic practices of institutions of higher learning and thus promotes a paradigm shift.

Program evaluation in competency-based medical education is more needed than ever [4]. Due to the Affordable Care Act (ACA) and other mandates, there is a greater emphasis on accountability and quality improvement in healthcare. The increasing pressure from the Center for Medicare and Medicaid Services (CMS) Hospital Compare ratings, and value-based payments are creating a "burning platform" (a metaphor used to relay a sense of serious urgency about the necessity of change despite the fear of the unknown consequences), highlighting the dire consequences of not changing. Hospitals may be subject to receiving reduced reimbursements if patient satisfaction scores are too low, and physicians can be penalized if Physician Quality Reporting System (PQRS) standards are not met.

There has been scant recent published literature on action learning in medical education, and although the concept and practice have been around for many years, the majority focused on nursing education and mathematics [5-10]. Topics addressed included improving surgical leadership, improving counseling skills in general practice, transitioning from clinicians to managers, virtual reflections of critical incidents, and increasing resident learning in a pediatric neurology clinic. In 2019 Zuber-Skerritt introduced a special type of action research called PALAR (participatory action learning and action research) that integrates lifelong action learning and participatory action research for researchers to improve their particular practice [11]. Jacobson and colleagues [A5] also endorse an action learning research approach to course improvement for a master’s in medical education degree and recommended an increased focus on action research, particularly education research for sustained course improvement such as has been done in the current study [12]. This study thus aimed to address the following research questions:

1. Did students demonstrate the KSAs needed to conduct master’s level formative and summative evaluations in health professions education?

2. Did the formative evaluation provide information to improve the current and future medical education assessment classes?

3. Did the action learning model enable students to experience some of the realities of conducting an evaluation?

This paper was published as a conference proceeding at the 14th Annual International Conference of Education, Research, and Innovation (ICERI) 2021. 

## Materials and methods

For this quality improvement study, the action learning methodology was applied in the Master of Science graduate degree program in Medical Education at the Nova Southeastern University (NSU) Dr. Kiran C. Patel College of Osteopathic Medicine (KPCOM) in Florida, United States. Most medical school courses complete summative evaluations when final grades are issued. They very rarely conduct serious formative evaluations, especially while the class is occurring. As a quality improvement study, this work was not subject to approval by the NSU Institutional Review Board. 

This study was a formative evaluation of a six (6) semester hour master’s level medical education assessment class course. A credit hour is defined as the number of hours spent in class per week over the duration of a semester. This time may be spent on discussions, readings and lectures, study and research, and assignments. The curriculum included the traditional knowledge, skills, and attitudes (KSAs) needed to conduct formative and summative evaluations in health education. In addition, students learned applicable quality improvement skills. Specifically, they learned how to identify and work with valid customer (student) requirements, how to create and improve processes, and how to collect and analyze process data. Students were taught the KSAs while conducting a formative evaluation of the class as their major project. They evaluated the class they were taking and could reflect on the experience.

**Figure 1:**
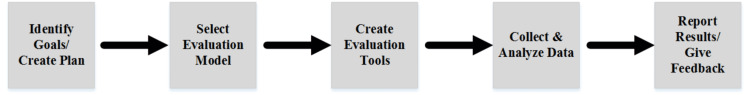
Evaluation Model

The course incorporated both the Bloom levels of learning and Kirkpatrick training evaluation models and addressed the following road map or process (Figure 1):

Study design and participants

Students were taught the medical education evaluation knowledge, skills, and attitudes (KSAs). Through this, they experienced the process of performing a formative evaluation. Nine students were enrolled in the course and participated; all were healthcare professionals (e.g., medical school faculty, dental school faculty, simulation lab managers). The students formed two groups of action learning sets. The class was delivered as a hybrid class (face-to-face and online instruction). The first and last sessions were held on-site, and the middle 14 sessions were held online. Students also had numerous supplemental calls and meetings with the instructor.

The course was mostly organized in traditional ways with a syllabus, rubrics, and assignment instructions. Some things were purposely left “vague” to introduce some of the “messiness” inherent in most evaluation projects. Finally, not all student questions were immediately answered. This allowed them to generate their own answers.

The data used for the formative evaluation were student-generated. Table 1 depicts the activities that produced the sources of data utilized for this study. Data collected from this cohort are reported as descriptive statistics and inferences based on non-parametric statistical analyses that seek to describe the results likely to be obtained from the population of similar cohorts of students. Rubrics were used to help operationalize the grading criteria. 

**Table 1:** Types of Data

Data Type	Frequency
Pretest/post-test results	once
Quiz results	5 quizzes
Weekly student feedback surveys (through last online class)	12 surveys
Class attendance	15 weeks
Class participation activity reports	13 weeks
Time students spend on class (from logs)	13 weeks
Assignment grades	15 assignments

The pre-and post-tests were alternate forms; both contained the same number, type, and difficulty of questions. Test and quiz results were coded to facilitate analysis. For example, tests results could be 2 = correct, 1 = partially correct, and 0 = incorrect.

During the first onsite class, students were asked to identify what they required from the instructor and the class. Input from students was used to develop the feedback survey. When completing the weekly surveys, students were tasked with writing up to three positive and three negative comments (plusses and deltas). Students were also asked to rate the importance of each of the survey items. The first three surveys were pencil and paper, and the remaining ones were online. This helped the students experience the two different methods.

Additional student reflections on the KSAs were solicited during every class. These ongoing ideas and opinions provided ideas for improving the course. Students also offered regular explanations of the feedback survey results so that the data could be better understood.

Assignments were graded, but they were designed to promote mastery of the skills being taught. Students were allowed multiple attempts to correct their submissions after receiving feedback from the instructor.

The five quizzes were open books because the instructor wanted the students to read and review the books and PowerPoint presentations. These assignment and quiz grades were uniformly high. Rare errors occurred when the students were asked to analyze and/or apply concepts.

## Results

The results related to the three-course goals were very positive. The KSAs were all mastered, course improvements were realized, and the students experienced a real formative evaluation. 

Pre- and Post-tests

**Figure 2:**
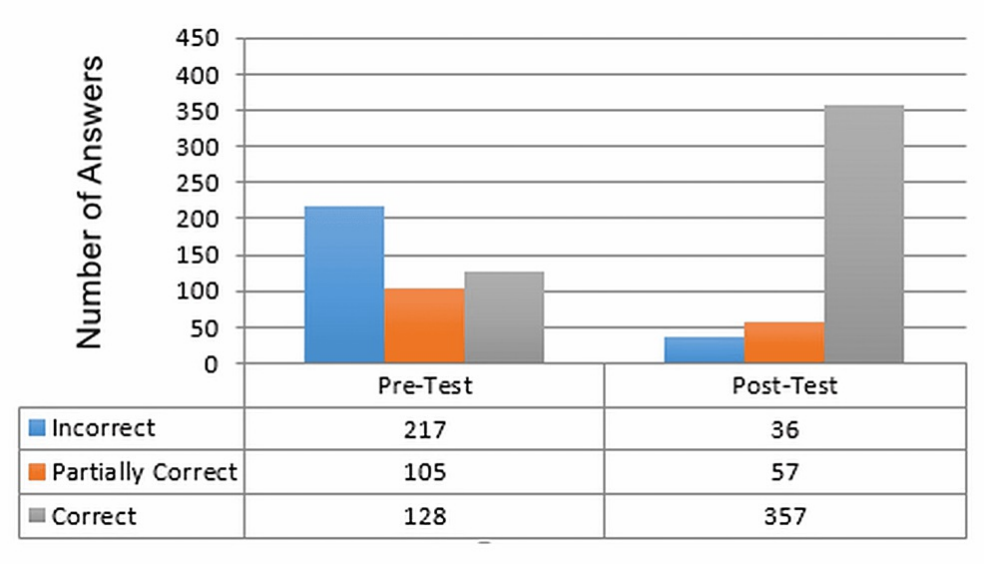
Pre- and Post-test Results

Figure 2 reports results that show major improvements from the pre-to the post-test. For example, there were 128 correct pretest answers and 357 correct answers on the post-test. The pre-and post-test results were further analyzed using the one-sample sign test for medians because the test assumptions for the type of data and distribution were met. This inferential test was used because it could help determine significance levels for inferring the results of this education evaluation class to a larger and similar population of the graduates in the current sample. 

The one-sample sign test was used to evaluate the median of the difference between the pre-and post-test groups. To do the sign test for each question for each student, the pretest result is subtracted from the posttest result. If the post-test result is greater than the pretest result this leads to a “+” contribution, while a pretest result that is greater than a post-test result leads to a “-” (negative) contribution. The results of this subtraction of each pre-and post-pair created one sample of either a 2,1,0, -1, or -2. Scores of “1” and “2” would be included in the sign test as a “+” while scores of “-1” and “-2” would have a negative contribution “-” on the sign test. Cases where there was no difference between the pretest and post-test results would yield a score of “0”, which does not contribute to the sign test. In a sign test, it is assumed that the distribution of positive “+” and negative “-” contributions are equal, so there are not statistically more positive “+” or negative “-” results if there is no difference in the pretest and post-test results.

In Table 2, the post-test results for all students combined revealed that 277 of the 450 (61.6%) data points were greater than zero. A total of 139 data points showed no improvement and 34 results were worse than the pretest. 

**Table 2:** One Sample Sign Test for Medians* * H0: Median = 0 and Ha: Median > 0

Results:	Post – Pre
Count (N)	450
Median	1
Points Below 0	34
Points Equal to 0	139
Points Above 0	277
Percent Above 0	62
P-Value (1-sided)	< 0.01
Assignment Grades	15 Assignments

The overall findings were then stratified by question type (see Table 3). “Analysis” questions refer to mostly data and statistics information. The “evaluation” items addressed formative and summative evaluation KSAs. “Quality” questions focus was on the process improvement body of knowledge, and the “questions” items refer to types of test questions such as multiple choice or short answer, etc. 

**Table 3:** One Sample Sign Test for Medians: Post-Pre by Question Type* * H0: Median = 0 and Ha: Median > 0

Type	Analysis	Evaluation	Quality	Questions
Count (N)	135	216	54	45
Median	1	1	1.50	1
Points Below 0	7	18	4	5
Points Equal to 0	36	76	10	17
Points Above 0	92	122	40	23
Percent Above 0	68	56	74	51
P-Value (1-sided)	< 0.01	< 0.01	< 0.01	< 0.05

There were significant improvements in all four of the question-type areas. The findings show that greater post-test gains occurred in the analysis and quality areas because they contained more new information for the students (see Table 4).

**Table 4:** One Sample Sign Test for Medians: Post-Pre by Student* * H0: Median = 0 and Ha: Median > 0

Students	S1	S2	S3	S4	S5	S6	S7	S8	S9
Count (N)	50	50	50	50	50	50	50	50	50
Median	1	2	2	0.50	2	1	1	1.50	0
Points Below 0	4	3	5	6	1	3	4	2	6
Points Equal to 0	18	11	9	19	6	17	15	13	31
Points Above 0	28	36	36	25	43	30	31	35	13
Percent Above 0	56	72	72	50	86	60	62	70	26
P-Value (2-sided)	< 0.01	< 0.01	< 0.01	< 0.01	< 0.01	< 0.01	< 0.01	< 0.01	0.1671

Further drill down revealed that eight of the nine students made statistically significant gains from the pre-to post. One student obtained 13 data points (26%) above zero. This was not significant progress at the .05 level.

A test item analysis was performed, and five of the 50 items had two or more incorrect post-test responses. All five of the questions dealt with descriptive statistics.

Assignments and quizzes 

The assignment scores ranged from 2.0 to 3.0 points, and the quizzes ranged from 4.75 to 6.0. These results were not comprehensively analyzed because they were all relatively high. Students submitted assignments multiple times until they mastered the skills based on the rubric criteria. They also obtained high quiz scores because they read the required texts and successfully searched for the correct answers. The few mistakes made were all due to poor application of higher-level learning such as analysis or comparison.

Student feedback surveys 

**Figure 3:**
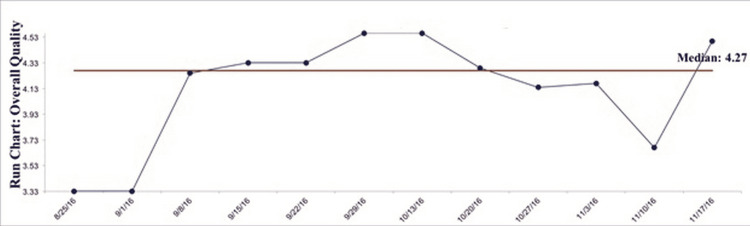
Weekly Student Quality Ratings

Student surveys were a major part of the formative evaluation. They were completed after every class instead of at the culmination, which is the most common practice. The results were discussed at the next session, and the instructor used them as “teachable moments” and suggestions for improvement. Students learned the relative strengths and weaknesses of survey methodologies. See Figure 3 line graph showing a perceived median overall quality rating of 4.27. The overall scores ranged from 3.33 to 4.53 on the 5.0-point scale.

**Figure 4:**
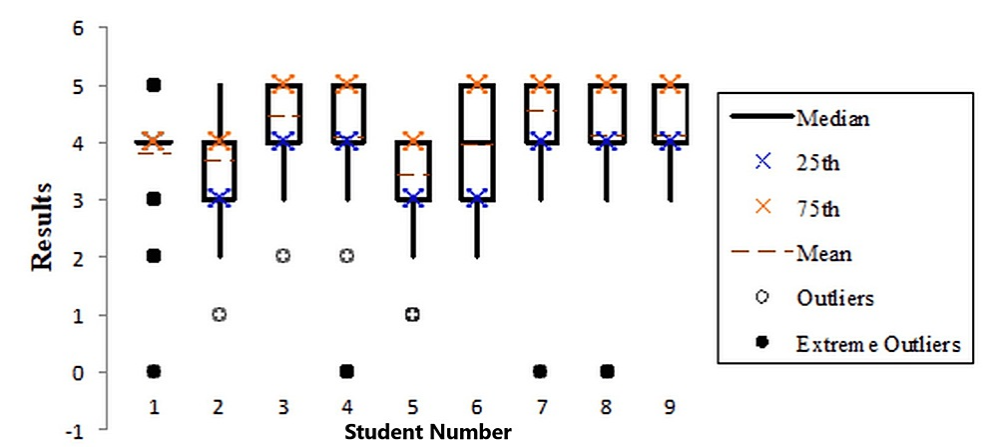
Feedback Survey Ratings by Student Number

Figure 4 illustrates that when survey results were stratified by the student, the boxplot indicates a distinct pattern. The first, second, and fifth students' survey scores were much lower than the other six students. These three students were also in one of the two groups. When the two lowest scores sets are removed, the overall average increases from 4.12 to 4.30. The students were also shown what effect removing two high scores can have on the mean. When the N is small, differences can have an impact. These two students attended every class, while some of the highest scorers did not. Note that "average" survey ratings should be examined with caution because this is ordinal data.

The Weighted Gaps results reported in Table 5 reveal some high positive scores that could be opportunities for improvement. Negative scores are less of a priority because satisfaction is higher than importance. Most evaluators do not consider both importance and satisfaction. This helps focus limited improvement resources. 

**Table 5:** Weighted Gaps

Survey Item	Importance	Satisfaction	Gap (I-S)	Weighted Gap (IxG)
Emphasis on Skills	4.56	4.2	0.36	1.64
Instructor Questioning	3.78	4.27	-0.49	-1.85
Instructor Explanations	4.89	4.05	0.84	4.11
Instructor Feedback	4.33	4.09	0.24	1.04
Instructor & Technology	3.56	3.87	-0.31	-1.1
Usefulness of Material	4.78	4.07	0.69	3.3
Practicality of Material	4.78	3.97	0.81	3.87
Assignment Directions	4.67	3.51	1.16	5.42
Length of Session	3.44	3.88	-0.44	-1.51
Overall Quality	4.67	4.11	0.56	2.62

The weighted gaps results reported in Table 5 reveal some high positive scores that could be opportunities for improvement. Negative scores are less of a priority because satisfaction is higher than importance. Most evaluators do not consider both importance and satisfaction. This helps focus limited improvement resources.

Student participation and study time 

The instructor tallied all student questions and comments during classes. This data did not correlate with student course results, but it did dramatically increase class interaction. Likewise, student study time did not improve grades probably because study methods and efficiency are more important factors. It was collected to provide variable (time) data for them to use on some assignments.

## Discussion

This section addresses each of the three research questions.

1. Did students demonstrate the KSAs needed to conduct master’s level formative and summative evaluations in health professions education?

The students improved their education evaluation knowledge and skills. Assignment and test score variation can be attributed to differences in abilities and effort. They also gained a greater appreciation of the importance of relevant “attitudes.” There was value in modeling and discussing attitudes and values associated with evaluations. The students became more aware of the connection between attitudes and behaviors. Too often, this is ignored, and most of the emphasis is placed on just knowledge and skills.

There was a tremendous amount of material to cover in this six-semester-hour class. This became evident when students were rushed to complete the formative evaluation project at the end of the term. This was exacerbated by the ordering of the learning to follow the evaluation roadmap presented above. More students struggled with the descriptive statistics material, and the more specific report writing information was presented last because it was the final step in the model. They could have started the formative evaluation data analysis earlier had they learned those skills earlier in the course.

Some students had trouble learning the statistical analysis software, and they relied on other team members to perform those analyses.

2. Did the formative evaluation provide information to improve the current and future medical education assessment classes?

The data were more meaningful to the students because it was their data. They were the subject matter "experts", and their insights aided analysis. This is especially true of their survey feedback data. They were also better able to connect their perceptions to actual events because the surveys were administered after each class.

The first two weeks produced the lowest scores because the students were generally overwhelmed and unsure of the complicated course requirements. The instructor made many modifications and spent time answering questions, and those changes led to higher ratings starting at week three. Results declined again during the statistics lessons and when the details of the final evaluation report format were discussed.

Feedback from students can be “painful” to receive if the students feel comfortable enough, to be honest. The students learned that there are preferred ways to “deliver a message.”

The student feedback survey results revealed that the most dissatisfaction was directed at assignment directions, instructor explanations, and the usefulness of the material. Students were most satisfied with the instructor's questioning and emphasis on skills.

Most of this feedback was beneficial, and the instructor made appropriate adjustments to the course as it went along. Mid-course corrections cannot occur when the surveys are only given at the end of the course.

3. Did the action learning model enable students to experience some of the realities of conducting an evaluation?

This experiential class was a new experience for most of the students, and it engendered some anxiety and “pushback.” Most of the students in this course have had traditional educational experiences and were unaccustomed to the action learning paradigm used by the instructor. This caused stress for the students, and they voiced their opinions through the weekly surveys as well as emails and phone calls. The expectation would be that the students would demonstrate professional behavior as well as appropriate communication. Unfortunately, some of the dialogue became heated and uncomfortable for other students.

There were two learning sets or groups in this class, and there were major differences in team make-up and leadership. One group became more dissatisfied, and much time was spent discussing their concerns.

Also, some of the students did not submit data and assignments on time, and this adversely effected their teams. This is typical of group projects in all work or academic settings; however, participants can be more carefully selected in a real-world work environment. For the formative evaluation, the data that was to be used was student-generated and dependent upon each student’s completion of the various assignments. There were times when assignments were not completed on time, which led to either a delay in data analysis or contributed to information not being usable. For instance, the post-test data were not available at the time of the formative evaluation submission. 

Another aspect of group work that was a challenge for the students was finding the best times to meet. Many of the students had other responsibilities aside from their work, including families, dependents, health issues, travel, and other obligations. In addition to having difficulties in finding meeting times, there were also issues with group members completing their other group responsibilities. The experiences and skills of the group members at times led to inequitable distribution of the workloads, yet all group members obtained the same grade.

Most students were interactive and participatory in the online sessions. This also increased after the instructor started measuring these behaviors. Some students resented the measuring of class participation, but they acknowledged the positive behavior changes. Interestingly, the most active students slowed down some and spoke less, while the less engaged spoke more.

The instructor was open and available to the students via email, text, phone calls, and in-person meetings, either individually or as groups. This afforded the students an extended ability to learn in this course outside of the weekly online sessions. However, not all students took advantage of these opportunities. 

Recommendations for medical education

This action learning approach for teaching medical education evaluation was very challenging, but it provided a much more realistic and rewarding educational experience. The students learned the basic KSAs needed to conduct formative and summative evaluations. They also gained a greater appreciation of the positive and negative aspects of using an experiential approach. Finally, the weekly formative surveys provided regular feedback that led to instructional improvements.

The following recommendations are offered:

1. Action learning courses should have three stages to them: pre, during, and post. In the “pre-phase,” students are introduced to the methodology. In the “during phase,” students live and experience the methodology. In the “post-phase,” students regroup with their instructor to process the experience and discuss challenges and opportunities for application. Such an arrangement will enhance the student’s learning and the instructor’s teaching experience.

2. Action learning is not a real prescriptive methodology, which could prove problematic for many health professionals because so much of their learning focuses on basic health science knowledge and related skill acquisition. With regards to medical education, action learning is best suited for students during the clinical phase of their education.

3. Action learning is impactful when used in small group settings. This methodology operates best when the group size does not exceed nine students. Large classes need to be divided into manageable groups.

4. Action-learning courses should not be sequenced as the very first course for students admitted into graduate medical education degree programs.

5. Greater emphasis should be placed on course prerequisites. Ideally, students enrolling in graduate-level evaluation courses should already possess knowledge of statistics, computer applications, and technology proficiency.

6. The order of the evaluation model presented in the Methods section above should be changed for instructional purposes. Students should be taught data analysis and report writing skills at the beginning so they could have “the end in mind” when they do the assignments and projects.

7. Instructors need to be realistic with group assignments. Project scoping is important because of student and time limitations. Student procrastination must be considered when due dates are determined. Also, this model should be reserved for more responsible students.

8. Team leaders need to be carefully selected, and they will need coaching from the instructor.

9. Finally, some possible content areas for effective use of action learning could include improving patient rounding, treatment planning, critical incident reviews, micro-system communication, patient flow, and patient safety.

Limitations

There were several limitations to this study. First, it was a small sample size, and results should be taken with caution as they cannot be generalized to other contexts. Second, the fixed time limits of this course were limited in scope as course semesters are finite. Third, important projects require the careful selection of team members, which was not possible.

## Conclusions

Findings from this review study indicate that an action learning approach for teaching a medical education evaluation course while challenging can be rewarding for graduate students as they learn basic KSA’s needed to conduct formative and summative evaluations. Students also found a greater appreciation for all aspects of using an experiential approach. Also, action learning in this context and small group settings may best be suited for students during the clinical phase of their education. Finally, content areas for effective use of action learning could include improving patient rounding, treatment planning, critical incident reviews, micro-system communication, patient flow, and patient safety. 
